# An Experimental Study on Defect Detection of Anchor Bolts Using Non-Destructive Testing Techniques

**DOI:** 10.3390/ma16134861

**Published:** 2023-07-06

**Authors:** Dongwoo Seo, Jaehwan Kim, Sangki Park

**Affiliations:** Department of Structural Engineering Research, Korea Institute of Civil Engineering and Building Technology, 283 Goyang-daero, Ilsanseo-gu, Goyang-si 10223, Gyeonggi-do, Republic of Korea; dwseo@kict.re.kr (D.S.);

**Keywords:** anchor bolt, non-destructive testing method, manual testing, PAUT

## Abstract

Anchor bolts are often used for fixing information boards, supports, and soundproof walls in various facilities. Corrosion of anchor bolts and fatigue cracks occur frequently due to the various external environments, and visual inspection and hammering inspection are mainly used. In visual inspection, it is difficult to confirm corrosion or fatigue cracks of anchor bolts in the area where foundations, nuts, and base plates are installed. Additionally, the hammering inspection is easily affected by the surrounding environment and the subjective reaction of the tester. Therefore, it is necessary to develop a method that can easily and accurately detect defects such as cracks and corrosion occurring in anchor bolts installed in road facilities using non-destructive testing techniques. In this paper, the possibility and reliability of anchor bolt defects such as corrosion and cracks were experimentally verified by applying ultrasonic inspection among non-destructive inspection techniques for anchor bolt maintenance.

## 1. Introduction

Anchor bolts are widely used for fixing and supporting various facilities. They are applied to fix or support road signs, information boards, sound barriers, etc. and are installed on bridges, highways, or roads. Generally, they are made of steel, but can also be made from aluminum, brass, and stainless steel. After installation, they are exposed to various external environments. For this reason, corrosion and deterioration of connected concrete facilities occur over time, resulting in decreased durability and deformation of the facilities.

For instance, in the case of bridges, contraction and expansion of the bridge structure due to sudden changes in external temperature, deformation of the structure such as creep and dry shrinkage due to the characteristics of concrete materials, and displacement due to the repeated action of vehicle traveling load occur [1]. To control the amount of expansion and contraction caused by these deformations, expansion joints are applied, and anchor bolts are used to connect the expansion joints and the bridge. The expansion joints installed on bridges are devices that block stress transmission between the structure and the structure, maintain the flatness of the bridge surface, and improve the drivability of vehicles and the safety of the structure [2].

Expansion joints and anchor bolts are subjected to damage and breakage due to external environmental factors such as salt water and the passage of overloaded vehicles that exceed the design load of the bridges, highways, and roads.

In 1983, the Mianus River bridge in Connecticut, U.S., was involved in an accident due to poor maintenance of expansion joints, resulting in three deaths. The 2007 collapse of the 35W Bridge in Minnesota, USA, was caused by a defective expansion joint, resulting in 13 deaths and dozens of injuries. This incident emphasized the importance of bridge structure safety and maintenance [3]. 

In South Korea, in June 2018, a protrusion of the expansion joint occurred due to the breakage of the anchor bolts for fixing the expansion joint installed on the bridge, and an accident occurred in which the protruding expansion joint damaged the tires of passing vehicles. In addition, there are many cases of broken anchor bolts, such as the fall of some expansion joints due to the breakage of the anchor bolts for fixing the expansion joint installed on one of the bridges of Busan in January 2022. When the anchor bolts installed on bridges break, the supporting structure shifts and deforms, posing a great risk to vehicles traveling on the bridge, and eventually causing major accidents such as vehicle damage and driver death, emphasizing the need for prevention [1].

In South Korea, non-destructive testing methods for anchor bolts are mainly visual and hammering inspections. A visual inspection is an inspection method that relies on the inspector’s eyesight for the part where the anchor bolts are installed, and it is difficult to secure the vision required to proceed with the inspection because the anchor bolts are buried in the foundation or blind areas can be existed due to the installation of the base plate. In the case of a hammering inspection, the inspection results may differ due to noise from the surrounding environment and the subjective judgment of the inspector, and even if the same inspector performs the inspection, it is somewhat difficult to perform an objective inspection due to external environmental factors. Therefore, it is necessary to develop an objective and quantitative inspection technology to identify defects in anchor bolts [4].

Non-destructive testing methods are those that use physical or chemical methods on the product or material to be tested, but do not cause destruction or deformation of the product or material, and check for internal or external defects. Among these non-destructive testing methods, ultrasonic testing is a technique that detects discontinuities caused by defects inside the object by using the phenomenon that ultrasonic waves travel straight through the interior of the object, reflecting and refracting. Recently, owing to the sophistication of ultrasonic equipment and the development of various inspection techniques, the detection performance of internal defects has been improved [5]. 

Ultrasonic non-destructive testing techniques include the Pulse Reflection method and Phased Array Ultrasonic Testing (PAUT). The Ultrasonic Pulse Reflection method, also known as the Ultrasonic Pulse Echo method, the most common method among ultrasonic detection methods, is a method that injects short-duration ultrasonic pulses into a product or material and receives reflections from internal defects to identify the location and size of the defect. While conventional ultrasonic testing utilizes two single devices for transmitting and receiving, phased array ultrasonic utilizes multiple devices. Unlike conventional methods, phased-array devices utilize ultrasonic waves with different refraction angles and superimposed ultrasonic waves, allowing the detection results to be visualized in a three-dimensional image [6,7,8,9].

Recently, Trtnik and Gams summarized their findings on non-destructive testing of cement-based materials. They noted that the application to cement-based materials is feasible and could be standardized with further research [10]. Marcantonio et al. noted that non-destructive testing techniques based on ultrasonic waves are very useful for inspecting fatigue, thermal, and corrosion damage inside materials [11].

Honarvar and Varvani-Farahani noted that an application of UT technology to the recent technology of additive manufacturing and studied its applicability to internal defect detection. The study was conducted by applying the existing method, UT, and the improved method, PAUT, etc. [12]. Kim et al. conducted a study on the application of non-destructive testing methods in the field of additive manufacturing (AM) to microscopic defects and microstructure variation, which are of interest in the AM field. They showed that non-destructive testing technologies can be used for quality inspection of products produced after AM [13].

Shi et al. conducted a study on the possibility of defect detection by automatically classifying non-destructive testing technologies resulting in images using convolutional neural networks (CNN) techniques [14]. Lee et al. conducted a study on the improvement of exploration results by combining PAUT results and artificial neural networks (ANN) techniques. Based on the 2D image results provided by PAUT, they applied the ANN technique to perform learning and studied the possibility of defect detection based on the results [15].

This study proposes an inspection method to identify defects in anchor bolts by utilizing the Ultrasonic Pulse Reflection method and phased array ultrasonic testing method, which are non-destructive testing methods using ultrasonic waves. For this purpose, anchor bolts were manufactured with various defects artificially imposed on them, and experiments were conducted using ultrasonic testing techniques. Based on the experimental results, it was confirmed that the method proposed in this study can detect defects inside anchor bolts.

## 2. Overview of Ultrasonic Non-Destructive Testing Methods

In general, ultrasound refers to the higher frequency components of sound, above 20 Hz to 20,000 Hz, which is the human audible frequency range. The frequency range of ultrasonic waves used in ultrasonic exploration is 0.2 to 25 MHz, and it utilizes the characteristics of ultrasonic waves, which are longer in wavelength than light and shorter in wavelength than ordinary radio waves, and that ordinary radio waves do not propagate inside the metal, while ultrasonic waves propagate inside the metal. If there are defects inside the test object, some ultrasonic waves are reflected, and this phenomenon can be used to investigate the presence, location, and size of defects [16,17].

**Figure 1:**
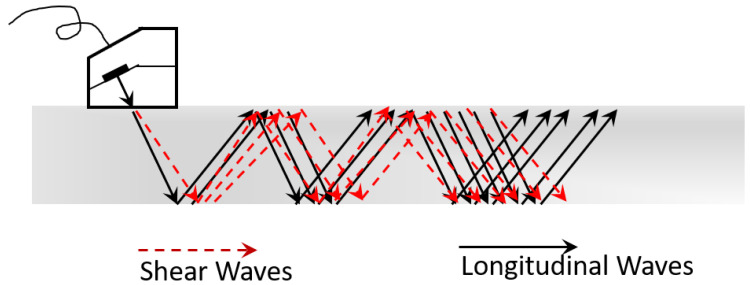
Principle of guided ultrasonic waves [17].

In this study, an ultrasonic inspection method that utilizes ultrasonic-guided waves that can travel long distances along complex and diverse object geometries was used. Unlike bulk waves, which typically travel through an infinite medium, guided ultrasonic waves have modes that exist in a wide frequency range, and most modes have dispersive properties, with propagation speeds varying with frequency and wall thickness. In guided ultrasound, each wave mode interferes with the other, so there are an infinite number of wave modes, and when traveling along the length of the pipe, interference occurs in the circumferential and thickness directions (see Figure 1). It is possible to obtain information about the corrosion state of anchor bolts by detecting changes in ultrasonic propagation speed when there are cracks and corrosion of anchor bolts due to corrosion in the section with guided ultrasound [7,9,17,18,19,20,21].

Therefore, non-destructive testing methods utilizing guided ultrasound can be performed on the entire structure at a fixed point without moving the probe, compared to localized testing methods using conventional longitudinal or transverse waves. In addition, the inspection of the structure can be performed without removing insulators or coatings, which is more time and economically efficient than conventional non-destructive testing techniques [16].

In the United States, non-destructive testing methods using ultrasonic waves are required to investigate the embedment depth, cracks, and other defects of hanger pins in bridges and anchor bolts in road facilities [12,13,14,15,16,17,18,19,20,21,22,23]. In Japan, ultrasonic testing methods are required to measure the length of anchor bolts using the pulse reflection method [24,25].

## 3. Anchor Bolts Defected Specimens

Anchor bolts mainly used in road facilities such as bridges are generally I, J, and L-shaped, and their types are M20, M24, and M30. Among them, M24 is commonly used, so the M24 type was selected to make experimental specimens. The KS regulation was applied when manufacturing the test specimen, and the washer was square [26,27]. The defects that occur in anchor bolts are mainly in the form of corrosion caused by exposure to the external environment and fatigue cracks caused by repeated loads such as vehicle movement, and these defects are generally found to occur mainly in the 50~150 mm section from the top of the anchor bolt. For this reason, when performing non-destructive testing for defect investigation, it was found that the shape of the anchor bolt is not related to the defect investigation. Therefore, in this study, a test specimen was manufactured for the type I shape among the three shapes of the anchor bolt. As shown in Table 1, a total of 16 specimens were selected, including 2 for reference without defects, 9 for corrosion defects, and 4 for crack defects.

**Figure 2:**
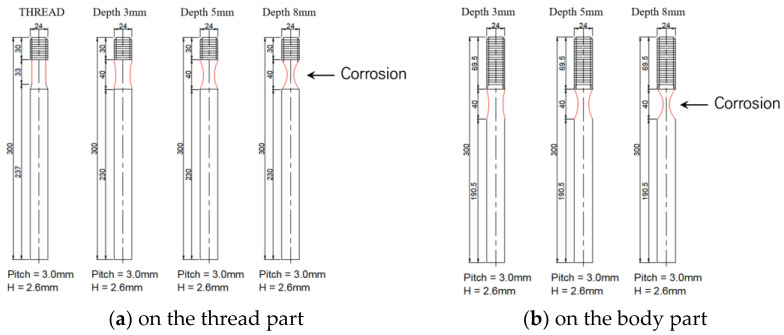

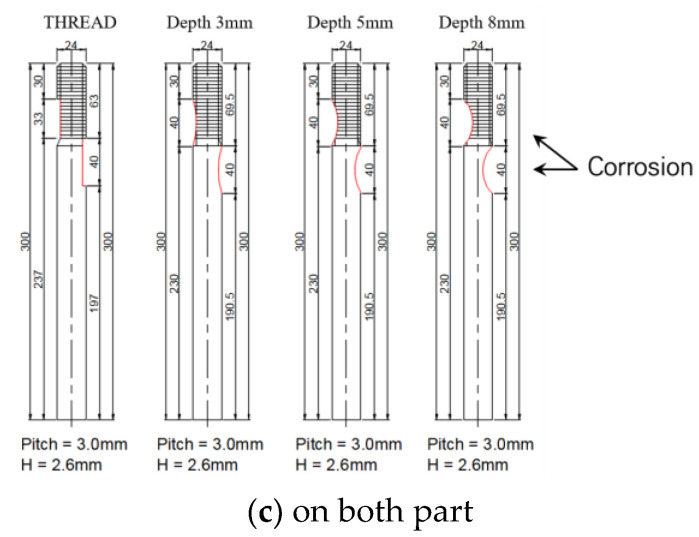
Anchor bolt specimens for corrosion case.

In the case of corrosion defects, the anchor bolts were divided into three categories: corrosion defects in the threaded part and the body part, corrosion defects in the threaded part only, corrosion defects in the body part only, and corrosion defects in both the threaded part and the body part. In the case of corrosion defects, the depth of corrosion was determined to be 3.0 mm, 5.0 mm, and 8.0 mm in three types. The type, size, and location of the defects in the anchor bolts were artificially created as shown in Figure 2.

**Figure 3:**
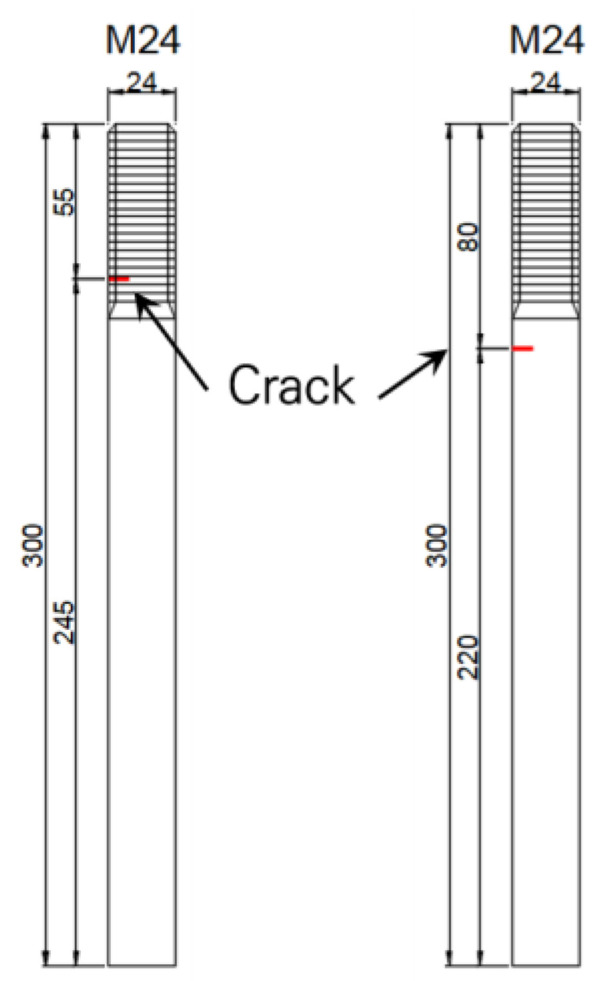
Anchor bolt specimens for crack case.

For crack defects, we categorized them into two types: crack defects on the threads of the anchor bolts and crack defects on the body of the anchor bolts. In the case of crack defects, the depth of the crack was determined to be 2.0 mm, 3.0 mm, 5.0 mm, and 7.0 mm for a total of four types. The type, size, and location of the defects in the anchor bolts were artificially created as shown in Figure 3.

**Figure 4:**
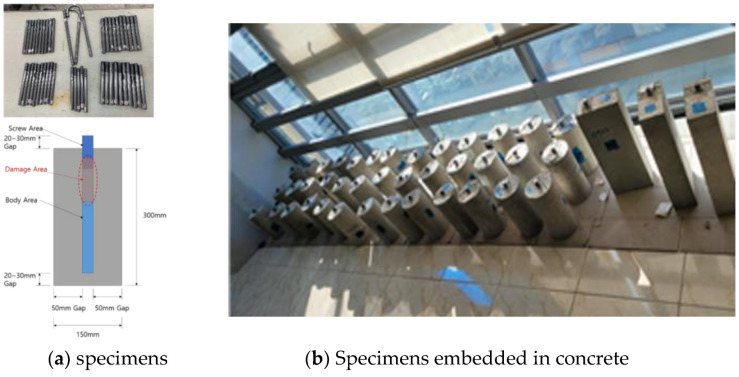
Anchor bolt specimens for testing.

In the real world, defective anchor bolts are embedded in concrete. To mimic this situation, the defective specimen was embedded in concrete to create a specimen that reflects the situation in the field as shown in Figure 4a. Figure 4b shows a defective specimen with artificially defective anchor bolts and a specimen embedded in concrete using this method.

## 4. Detect Anchor Bolt Defects through Field Experiments

Using the Pulse Reflection method and PAUT, the tests were conducted to detect defects such as corrosion and cracks in anchor bolts. The defects detection tests were conducted in two phases. The first stage was conducted before embedding in concrete, and anchor bolts with corrosion and crack defects were checked with the naked eye to determine the location and shape of the defects and detected using the pulse reflection method. In the second stage, defect detection was performed using the Pulse Reflection method and PAUT on the specimen after the anchor bolts were embedded in concrete.

### 4.1. Defects Detection Conditions Using Pulse Reflect Methods

**Figure 5:**
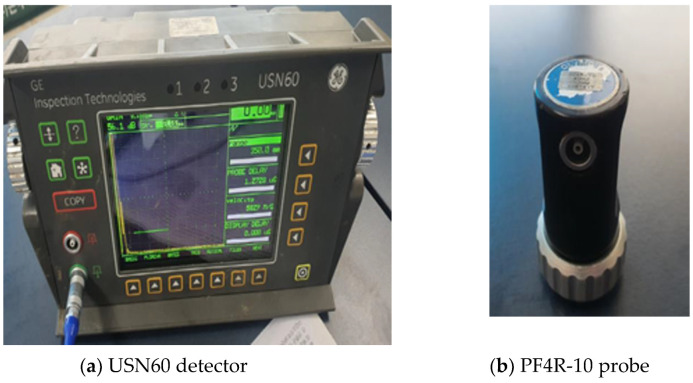
Testing equipment for The Pulse Reflect method.

For defect detection on the anchor bolt specimen, the pulse reflectography detector was a USN 60 from General Electric Company (GE Inspection Technologies, LP, Lewiston, PA, USA.) and the probe was a PF4R-10 from Olympus (Evident Korea Co., Seoul, Republic of Korea), and the longitudinal vertical defect detection method was applied. Figure 5 shows this equipment.

In the case of the anchor bolt to be inspected, the anchor bolt has a small area and a long length in the vertical direction. Therefore, when using the pulse reflection method, the longitudinal direction was set to the vertical direction, the sound speed was set to 5892 m/s, and the refraction angle was set to 0°. The ultrasonic energy was set from 100 to 1500 mm depending on the axial position, and the probe used a single 4 MHz oscillator with a diameter of 10 mm. The beam travel and sensitivity were calibrated against a 100 mm thick surface of the STB-A1. The readout was performed by manually spot-measuring the probe in the axial center direction to observe the presence or absence of a defect between the bottom line of the A Scan and the transmit pulse on the screen of the detector. To measure defects by the pulse reflection method, a defect-free anchor bolt specimen (300 mm long) was used to calibrate the detection reference sensitivity.

### 4.2. Defects Detection Conditions Using PAUT

**Figure 6:**
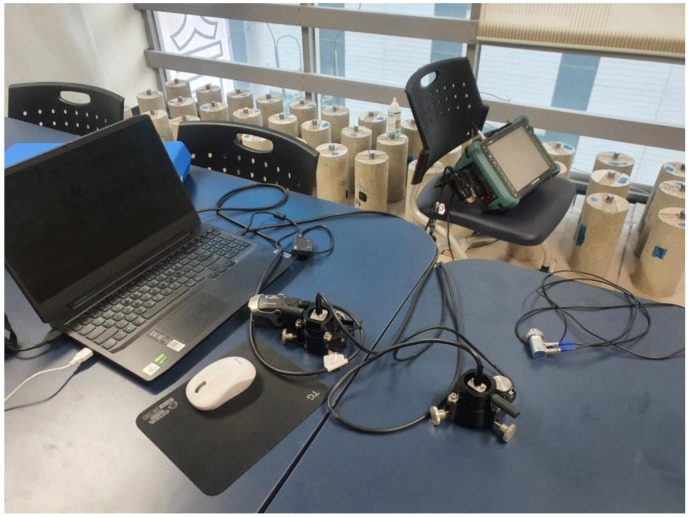
Testing equipment for PAUT.

The measurement system consists of a phased array defect detector, a jig, a computer, and an array probe. The phased array defect detector is an OMNI MX2 32/128P from Olympus (Evident Korea Co., Seoul, Republic of Korea), and the phased array defect detector excites ultrasonic waves that propagate in the voltages by applying a voltage to the array probe. The ultrasonic signal received by the array probe is input to the defect detector through the phased array probe and stored. The array probe is fabricated and installed in a jig, and the jig is manually rotated around its axis center. There are a variety of array probes available, but this study uses a relatively inexpensive linear array probe. Array probes other than linear array probes can also be used in this measurement system. Figure 6 shows the devices for PAUT.

Like the pulse reflection method, the phased array ultrasound method was based on the vertical direction, which is the longitudinal direction, and the steering function of the vibrator, which is an advantage of the phased array ultrasound method, was used to apply the focal law from −30° to 30° in a step of 1°, and the sound speed was set to 5900 m/s to perform the inspection.

The ultrasonic energy was set to Natural according to the axial position, the probe was a linear array probe with 32 oscillators at 5 MHz, and when receiving, the received waveform was focused to 50 mm or 100 mm from the axial position using the DDF (Dynamic Depth Focusing) function to improve the axial distance resolution. The beam travel and sensitivity were calibrated against a 100 mm thick face of the STB-A1. The readings were obtained by attaching a probe to the wedge, installing it in the jig, and using the jig to manually rotate the bolt 360° around its axial center to obtain A, S, and B scans to observe the presence or absence of flawed noses. The test was performed using a defect-free anchor bolt specimen (300 mm long) to calibrate the detection reference sensitivity so that all beams have the same sensitivity.

### 4.3. Experiment Results Using Pulse Reflection Methods

**Figure 7:**
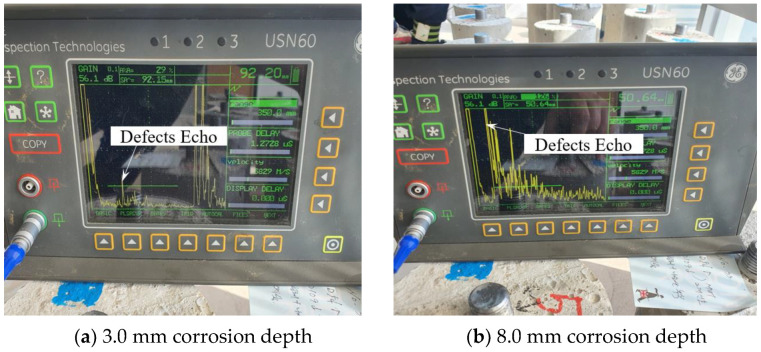
Result of detecting corrosion on the thread part case.

Using the pulse reflect method, an experiment was performed on a thread with corrosion (see Figure 7). The corrosion defects present in the threads are located between 30.0 mm and 70.0 mm, with the center of the corrosion defect located at 50.0 mm.

Figure 7a shows the experimental results for a corrosion depth of 3.0 mm. Figure 7b shows that when the depth of corrosion is 8.0 mm, the defect signal is detected at 50.64 mm, and it can be seen that it is possible to measure with high accuracy compared to the actual center of the defect at 50.0 mm. Comparing the results of 3.0 mm and 8.0 mm, it can be seen that the presence or absence of defects can be detected in the case of relatively small corrosion defects, and that the presence or absence of defects and even the location of defects can be detected relatively accurately in the case of relatively large defects.

**Figure 8:**
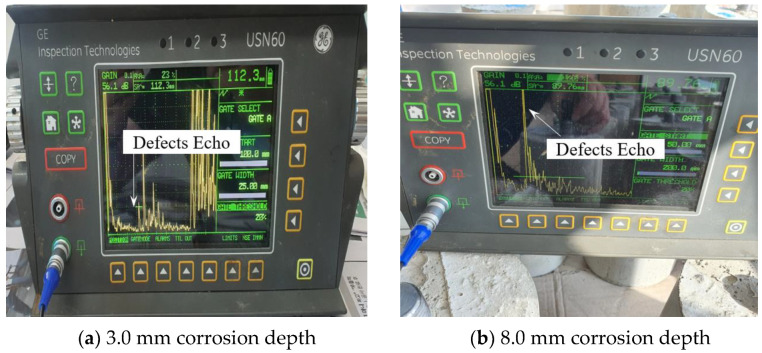
Result of detecting corrosion on the body part case.

Experiments were conducted on the case where corrosion is present on the body. The corrosion defects on the body are located between 69.50 mm and 109.5 mm, and the center of the corrosion defect is located at 89.5 mm. Figure 8a shows the experimental results for a corrosion depth of 3.0 mm, and the defect signal is detected at 112.3 mm. Figure 8b shows that when the corrosion depth is 8.0 mm, the defect signal is detected at 89.76 mm, and it can be seen that it is possible to measure with high accuracy compared to the actual center of the defect at 89.5 mm.

Experiments were performed on a case with corrosion on both the threads and the body. The corrosion defects on the threads are between 30.00 mm and 70.00 mm, with the center of the corrosion defect at 50.0 mm. The corrosion defects on the body are located between 69.50 mm and 109.5 mm, and the center of the corrosion defect is located at 89.5 mm. Inspection of a relatively deep specimen with a depth of corrosion of 3.0 mm shows that there is corrosion on both the thread and the body, but the physical inspection shows that there is only one corrosion, indicating that the accuracy is low for shallow depths.

**Figure 9:**
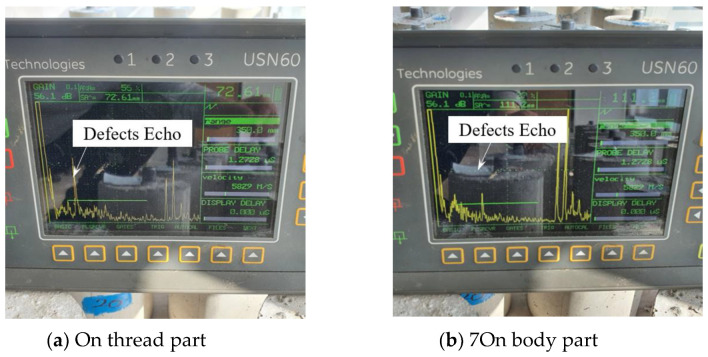
Result of detecting corrosion in both cases.

Figure 9 shows the experimental results for a case where the depth of corrosion is 8.0 mm. In Figure 9a, a corrosion defect on the thread is detected, and the location of the corrosion is determined to be 72.61 mm, and in Figure 9b, a corrosion defect on the body is detected, and the location of the corrosion is determined to be 111.2 mm.

**Figure 10:**
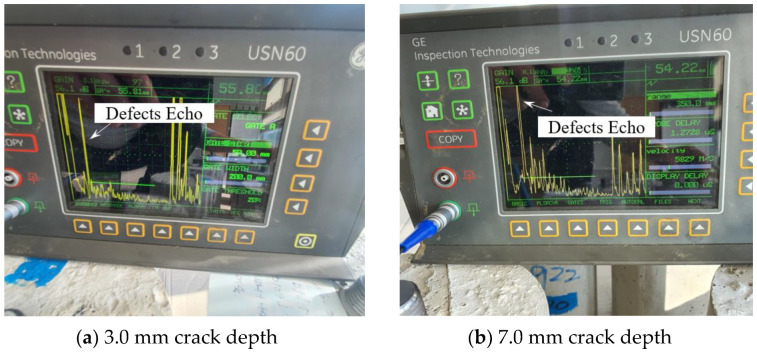
Result of detecting a crack in the thread part case.

Experiments were conducted with cracks in the thread or body. The crack defect in the thread is present at 55.0 mm. Figure 10a shows the experimental results for a crack depth of 3.0 mm, and the defect signal is detected at 55.80 mm. Figure 10b shows that when the corrosion depth is 7.0 mm, the defect signal is detected at 54.22 mm, and it can be seen that it can be measured with high accuracy compared to the actual defect location of 55.0 mm. In the case of crack defects, it can be seen that the presence of the defect and even the location of the defect can be detected relatively accurately.

**Figure 11:**
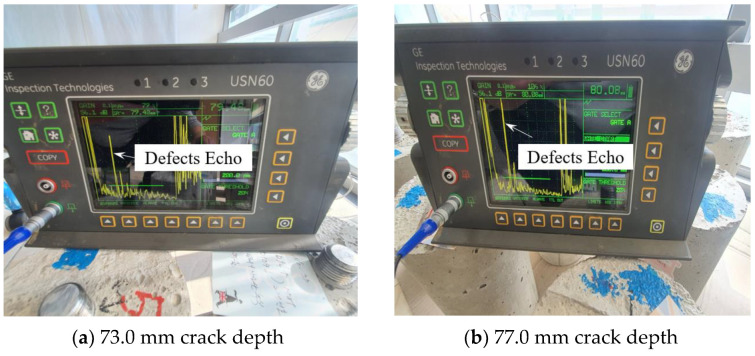
Result of detecting a crack in the body part case.

The crack defect in the body is present at 80.0 mm. Figure 11a shows the experimental results for a crack depth of 3.0 mm, and the defect signal is detected at 79.48 mm. Figure 11b shows that when the crack depth is 7.0 mm, the defect signal is detected at 80.08 mm, and it can be measured with high accuracy when compared to the actual defect location of 80.0 mm.

### 4.4. Experimental Results Using PAUT

**Figure 12:**
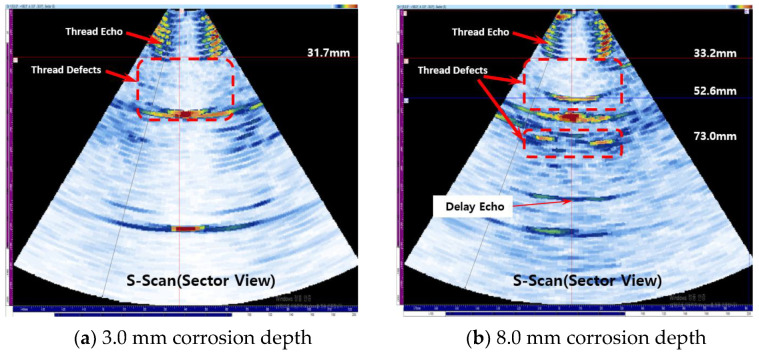
Result of detecting corrosion on thread part case.

Using phased array ultrasound, experiments were performed on the case of corrosion in the threads. Figure 12a shows the experimental results for a corrosion depth of 3.0 mm. Figure 12b shows that when the depth of corrosion is 8.0 mm, the defect signal is detected at 33.2 mm and 73.0 mm, and it can be measured with high accuracy compared to the actual defect start point of 30 mm and end point of 70.0 mm. Comparing the results of 3.0 mm and 8.0 mm, it can be seen that it is possible to detect the presence and location of defects in the case of relatively small corrosion defects, and it can be seen that the presence and location of defects and the range of defects can be detected relatively accurately in the case of relatively large defects.

**Figure 13:**
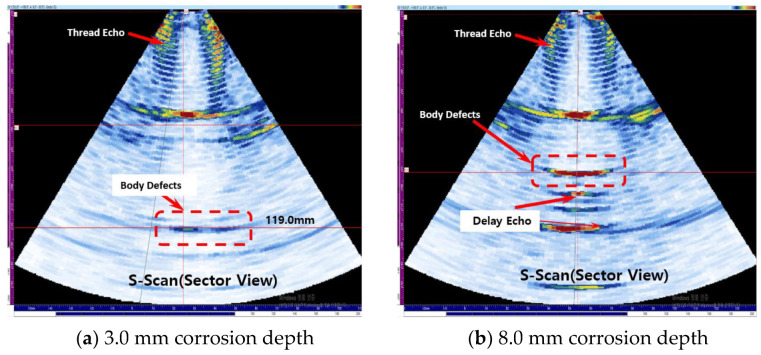
Result of detecting corrosion on body part case.

Experiments were conducted on the case where corrosion is present on the body. The corrosion defects on the body are located between 69.50 mm and 109.5 mm, and the center of the corrosion defect is located at 89.5 mm. Figure 13a shows the experimental results for a corrosion depth of 3.0 mm, and the defect signal is detected at 119.0 mm, but the signal is measured very weakly, so it is difficult to judge it as a defect. In Figure 13b, it can be seen that when the depth of corrosion is 8.0 mm, the defect signal is detected at 90.6 mm, and it can be measured with high accuracy when compared to the actual center of the defect at 89.5 mm.

The test was performed on a relatively deep specimen with a depth of 3.0 mm, which showed corrosion on both the thread and the body, but the actual test showed only one corrosion, indicating that the accuracy is low at shallow depths.

**Figure 14:**
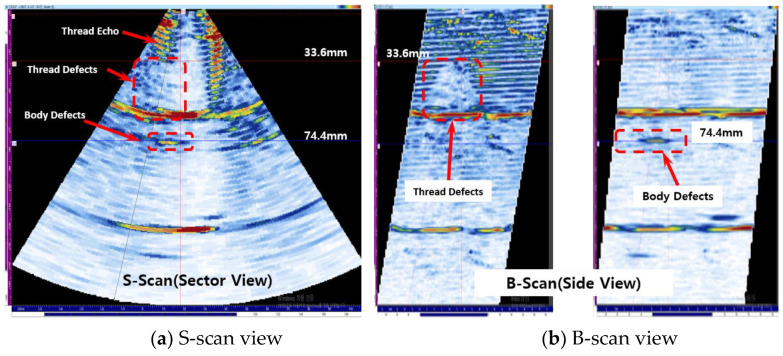
Result of detecting corrosion on both thread and body part case.

Figure 14 shows the experimental results for a corrosion depth of 8.00 mm. A corrosion defect was detected on the thread, and the location of the corrosion was determined to be 33.60 mm, and a corrosion defect was detected on the body, and the location of the corrosion was determined to be 74.40 mm.

**Figure 15:**
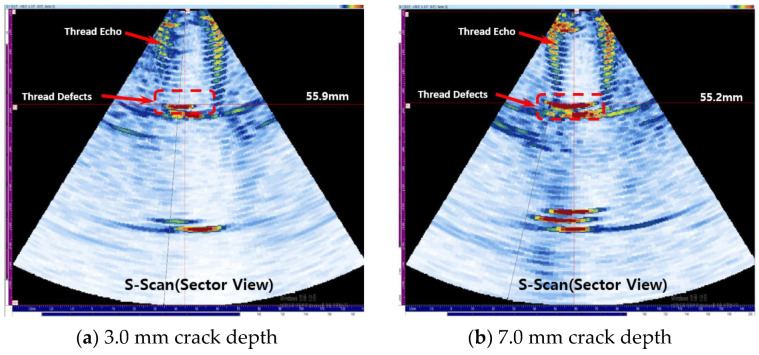
Result of detecting crack in thread part case.

Experiments were conducted with cracks in the thread or body. The crack defect in the thread is present at 55.0 mm. Figure 15a shows the experimental results for a crack depth of 3.0 mm, and the defect signal is detected at 55.90 mm. Figure 15b shows that when the corrosion depth is 7.0 mm, the defect signal is detected at 55.20 mm, and it can be seen that it can be measured with high accuracy compared to the actual defect location of 55.0 mm. In the case of crack defects, it can be seen that the presence of a defect and even the location of the defect can be detected relatively accurately.

**Figure 16:**
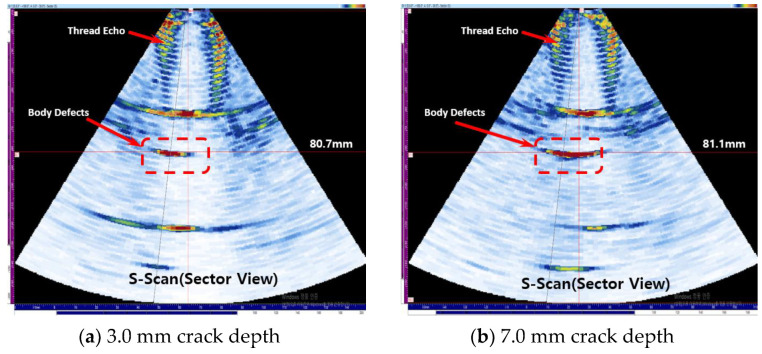
Result of detecting a crack in body part case.

The crack defect in the body is present at 80.0 mm. Figure 16a shows the experimental results for a crack depth of 3.0 mm, and the defect signal is detected at 80.70 mm. Figure 16b shows that for a crack depth of 7.0 mm, the defect signal is detected at 81.10 mm, and it can be measured with high accuracy when compared to the actual defect location of 80.0 mm.

The experimental results of the two cases are summarized in Table 2 and Table 3. From Table 2, the ratio of detecting corrosion is about 100% for the Pulse Reflection method and 90% for PAUT, roughly. In finding the location of corrosion, the accuracy of the Pulse Reflection method is slightly higher than that of PAUT based on the defect location, which is the central location of the corrosion defect range. However, it can be seen that PAUT is more accurate in the detection of defect location except for the case of not detection.

From Table 3, the ratio of detecting cracks is about 100% and PAUT was more accurate than Pulse Reflection in finding the location of cracks. However, detecting and finding the location of the corrosion case, the corrosion has some sort of range.

## 5. Conclusions

In this study, experiments were conducted on corrosion and cracking, which are types of defects that can occur in anchor bolts, and detection performance tests were conducted using the Pulse Reflection method and PAUT. To simulate the conditions in which anchor bolts are actually installed, the specimens were embedded in concrete, and the following conclusions were drawn as a result of the performance test.

(1) For corrosion

For thread-wide corrosion, the results of PAUT using S-Scan rather than pulse reflection methods are more reliable because the location and size of the corrosion can be determined. In the case of body corrosion, both non-destructive testing methods detect a signal of the defect, but the deeper the cross-sectional depth of the corrosion, the more accurate the location of the defects. For thread and body corrosion, both non-destructive testing methods detect a signal of the defect, but the deeper the cross-sectional depth of the corrosion (in the case of 8.0 mm), the more accurate the location of the defect can be determined.

(2) For cracks

In the case of thread cracks or body cracks, the pulse reflection method could only identify the location of the crack. Since the phased array ultrasound method can confirm the location and size of the crack, it was found that the phased array ultrasound method is more reliable than the pulse reflection method.

Overall, as a result of the experiments conducted on anchor bolts using ultrasonic testing for defects caused by corrosion and cracking, it is judged that the detection performance is low for cases where the size of the defect is relatively small, and in other cases, it is possible to detect the size, location, and extent of the defect. Furthermore, it is believed that the low detection rate for relatively small defects is related to the range of available frequencies of the probe, and if the frequency range is diversified by utilizing various probe types through further research, it is believed that sufficient detection is possible.

## Figures and Tables

**Table 1:** Experiment specimens for anchor bolt damage detection.

Damage Type	Quantity	Notes
No damage	2	I-types, 300 mm length
Corrosion	9	3 types * 3 = 9
Crack	4	2 types * 2 = 4
Overall	16	

**Table 2:** Summary of experimental results of detecting corrosion.

Type	Specimen ID.	Damage ^1^ Depth (mm)	Predictions (mm)	
			Pulse Reflect	PAUT
Thread Only	TCF-3.0	30~70	92.20	31.70
	TCF-5.0	30~70	70.74	32.50
	TCF-8.0	30~70	50.64	33.20, 52.60
Body Only	BCF-3.0	69.5~109.5	130.8	N/A
	BCF-5.0	69.5~109.5	109.0	110.30
	BCF-8.0	69.5~109.5	89.76	90.60
Thread & Body	MCP-3.0	30~70	60.53	34.40
		40.0~103.0		
	MCP-5.0	30~70	61.17	34.00
		69.5~109.5		
	MCP-8.0	30~70	72.61	33.60
		69.5~109.5	111.2	74.40

^1^ For the corrosion case, the damage appears as a range as shown in Figure 2.

**Table 3:** Summary of experimental results of detecting crack.

Type	Specimen ID.	Damage Depth (mm)	Predictions (mm)	
			Pulse Reflect	PAUT
Thread Only	TF-3.0	55.00	55.80	55.90
	TF-7.0	55.00	54.20	55.20
Body Only	BCF-3.0	80.00	79.50	80.70
	BCF-8.0	80.00	80.10	81.10

## Data Availability

Not applicable.
